# Supplementary material for: Towards practical and robust DNA-based data archiving using the yin–yang codec system
Source: Nat Comput Sci. 2022 Apr 25;2(4):234–42. doi: 10.1038/s43588-022-00231-2 (PMC10766522; doi:10.1038/s43588-022-00231-2)
Supplement: Supplementary file 1 — Supplementary Sections 1–3, Figs. 1–6 and Tables 1–7. [file 43588_2022_231_MOESM1_ESM.pdf]

nature portfolio

https://doi.org/10.1038/s43588-022-00231-2

Supplementary information

Towards practical and robust DNA-based data archiving using the yin–yang codec system

In the format provided by the authors and unedited

Supplementary Information includes:

Supplementary Section 1: Detailed workflow and features of YYC system

Supplementary Section 2: Quantitative analysis

Supplementary Section 3: Command line steps to encode and decode the data

Supplementary Figures

Supplementary Figure 1. The transcoding illustration using one of the 1536 coding schemes of YYC.

Supplementary Figure 2. Information density evaluation of 1536 YYC coding schemes using 1G data collection.

Supplementary Figure 3. Heatmap of average Hamming distance between sequences generated by 1,536 coding schemes.

Supplementary Figure 4. Statistics of average iteration runs of 1 GB data encoding using all 1,536 rules.

Supplementary Figure 5. The simulation analysis and experimental validation of YYC's data recovery rate in the presence of varying gradient of DNA sequence loss.

Supplementary Figure 6. Evaluation of the influence of sequencing depth on sequence error rate and sequence loss rate for *in vitro* storage validation.

Supplementary Tables

Supplementary Table 1. The effect on compatibility of generated DNA sequence using different coding strategies for specific binary data patterns.

Supplementary Table 2. Performance of 1536 coding schemes on binary segments with different 0/1 ratio.

Supplementary Table 3. The estimation of iteration runs and corresponding information density by transcoding 10 different types/formats of files.

Supplementary Table 4. The estimation of information density and logical redundancy by DNA Fountain and YYC to varying file types.

Supplementary Table 5. The in-silico simulation of the data recovery rate in the context of a gradient of DNA sequence loss.

Supplementary Table 6. Minimum redundancy required for successful data retrieval with different bitmap images.

Supplementary Table 7. Error analysis of data coding DNA in pRS416 for in vivo storage demonstration.

References

Supplementary Section 1: Detailed workflow and features of YYC system

The pipeline for the YYC system includes three main steps: segmentation, incorporation, and validity-screening (Figure 1b). For segmentation, the binary information is extracted from a source file and partitioned into multiple segments according to requirements. Binary indices are assigned and added to each segment to form a new one (information + index), and thus a pool of binary segments of identical length are then obtained. In the incorporation step, two segments are selected randomly to be incorporated into one DNA sequence according to the YYC algorithm. For the validity-screening in the last step, the incorporated sequence will be subjected to screening against pre-set constraints, including GC content, maximal homopolymer length, the secondary structure free energy, etc., and only sequences that meet set criteria will be considered as valid sequences. If the DNA sequence fails to meet these criteria, it will be discarded. The second selected binary segment (using "Yin" rule) will be put back into the pool and the iteration process will be activated: another randomly generated binary segment from the library will be added for incorporation with the first selected segment (using "Yang" rule) until a valid DNA sequence is generated. Considering for some digital information with particular data pattern (i.e., extreme ratio of 0/1), generating a valid DNA sequence with biocompatible features might be challenging since multiple runs of binary segment incorporation would be required. Thus, we evaluated the possibility of generating valid sequence by YYC coding schemes under varying range of 0/1 ratio from 0% to 50%. By simulating the incorporation of all binary sequences of one byte segment (8 bits), our result shows that the possibility of generating valid sequence (i.e., the number of coding schemes in the 1536 collection that can generate valid sequence) significantly drop from 100% to 49.7% in the presence of binary sequence with the 0/1 ratio lower than 20% (Supplementary Table 2). It implies that with the binary segment contains extreme 0/1 ratio, the encoding process will be significantly time consuming. To avoid the circumstance, we established a pre-screening process to identify 0/1 biased binary segments (0/1 ratio ⩽ 20%), to which a "firewall" is set for the upper limit of iteration runs of incorporation at 100 (Supplementary Table 3). For segments that fail to pass the process, a "pseudo" binary segment with random 0/1 but in balanced ratio will be introduced to allow the generation of a valid DNA sequence. By analyzing the

number of iteration runs of encoding files, our result shows that 65.04% of all segments can be successfully paired and pass the screening at first attempt. Only less than 0.002% of segments fail to generate a valid sequence after 100 iterations. Even in the worst case with most unbalanced data pattern we observed, the additional information added to the source file for successful transcoding accounts for only 19.25% of the original file size, with the average number of trails at ~7 and information density at ~1.45 bits/nt (Supplementary Table 3). It suggests that the YYC would not cost a large encode-time overheads (Supplementary Figure 4). Since very limited “pseudo” binary segments would be added into the source files, the information density can be well-maintained at a relatively high level for YYC.

Supplementary Section 2: Quantitative analysis

According to the Shannon information entropy [1], the information density (d_{THEORY}) of DNA-based data storage can be defined as

$$d_{\mathrm{THEORY}} = \frac{\log_2 C_{\mathrm{DNA}}}{l_{\mathrm{DNA}}}, \tag{1}$$

where l_{DNA} is the number of nucleotides of a DNA sequence and C_{DNA} refers to the amount of available DNA sequences of length l_{DNA}. In the condition without any constraints introduced, C_{DNA} equals to $4^{l_{\mathrm{DNA}}}$ and d_{THEORY} is 2.

Constraint coding in DNA-based data storage

In practice, considering the compatibility with DNA synthesis and sequencing technologies, maximum homopolymer runs (HOMO) and GC content bias (GCBIAS) are two most critical biochemical constraints for transcoding.

Let C_{DNA}^{v} be the amount of valid DNA sequences of length l_{DNA} under the constraints of HOMO and GCBIAS. The influence of HOMO on C_{DNA}^{v} can be represented as:

$$C_{\mathrm{DNA}}^{v} = \lambda^{l_{\mathrm{DNA}}}, \tag{2}$$

where λ is the largest real root [2] of the equation:

$$x^{\mathrm{HOMO}} - \sum_{i=1}^{\mathrm{HOMO}-1} 3x^{i} = 0. \tag{3}$$

Simply, combining equation (1) and (2), the constraint of maximum homopolymer runs (f_{HOMO}) is:

$$f_{\mathrm{HOMO}} = 2 - \log_2 \lambda. \tag{4}$$

Similarly, the constraints of GCBIAS on C_{DNA}^{v} can be represented as:

$$C_{\mathrm{DNA}}^{v} = \sum_{l=\left\lceil(\frac{1}{2}-\mathrm{GCBIAS})\times l_{\mathrm{DNA}}\right\rceil}^{\left\lfloor(\frac{1}{2}+\mathrm{GCBIAS})\times l_{\mathrm{DNA}}\right\rfloor} \binom{l_{\mathrm{DNA}}}{l} \times 2^{\,l_{\mathrm{DNA}}}, \tag{5}$$

where $\binom{l_{\mathrm{DNA}}}{l}$ represents the combination number of l selected from l_{DNA} and 2 refers to two set of nucleotides which affect the GC ratio. The GC content of sequence is within the interval of $[\frac{1}{2} + \mathrm{GCBIAS}, \frac{1}{2} - \mathrm{GCBIAS}]$, thus the lower bound is the ceiling of $(\frac{1}{2} - \mathrm{GCBIAS}) \times l_{\mathrm{DNA}}$, while upper bound is the floor of $(\frac{1}{2} + \mathrm{GCBIAS}) \times l_{\mathrm{DNA}}$. Combining equation (1) and (5), the constraint of GC content bias (f_{GCBIAS}) is:

$$f_{\mathrm{GCBIAS}} = 1 - \frac{\log_2 \sum_{l=\left\lceil\left(\frac{1}{2}-\mathrm{GCBIAS}\right)\times l_{\mathrm{DNA}}\right\rceil}^{\left\lfloor\left(\frac{1}{2}+\mathrm{GCBIAS}\right)\times l_{\mathrm{DNA}}\right\rfloor} \binom{l_{\mathrm{DNA}}}{l}}{l_{\mathrm{DNA}}}. \tag{6}$$

Considering index used in DNA-based data storage because of its unordered nature, let n_{BIN} be the number of binary segments and l_{BIN} be the length of binary segment in practice. Their impact on the theoretical upper bound of information density can be represented as:

$$d_{\mathrm{THEORY}} = 2 \times \frac{l_{\mathrm{BIN}} - \lceil \log_2 n_{\mathrm{BIN}} \rceil}{l_{\mathrm{BIN}}}, \tag{7}$$

where $\log_2 n_{\mathrm{BIN}}$ represents the combination number of all kinds of possible index. Simply, combining the equation (1) and (7), the constraint of index (f_{INDEX}) is

$$f_{\mathrm{INDEX}} = \frac{\lceil \log_2 n_{\mathrm{BIN}} \rceil}{l_{\mathrm{BIN}}}. \tag{8}$$

Summing up the above, the theoretical upper bound of information density (d_{THEORY}) can be represented. The influence of these constraints (f_{HOMO}, f_{GCBIAS}, and f_{INDEX}) on d_{THEORY} is usually overlapped. According to set theory [3], d_{THEORY} can be solitarily influenced by most influential constraints, that is:

$$d_{\mathrm{THEORY}} \leq 2 - \max\{f_{\mathrm{HOMO}}, f_{\mathrm{GCBIAS}}, f_{\mathrm{INDEX}}\}. \tag{9}$$

Assuming that the influence of all constraints on d_t is disjoint, there must be an existing coding scheme as the baseline, the baseline information density (d_{BASELINE})

of which satisfies that:

$$d_{\mathrm{BASELINE}} = 2 - \mathrm{sum}\{f_{\mathrm{HOMO}}, f_{\mathrm{GCBIAS}}, f_{\mathrm{INDEX}}\}. \tag{10}$$

Calculation of Theoretical information density of YYC

By applying the above equations, it is easy to evaluate the difference between actual information density interval and theoretical upper bound for a transcoding algorithm. Equivalently, the actual information density of YYC (d_{ACTUAL}) can be represented as

$$d_{\mathrm{ACTUAL}} = \frac{n_{\mathrm{BIN}} \times (l_{\mathrm{DNA}} - l_{\mathrm{INDEX}})}{\frac{(n_{\mathrm{BIN}} + n_{\mathrm{BIN}}^{a})}{2} \times l_{\mathrm{DNA}}}. \tag{11}$$

where n_{BIN} represents the original number of binary segments obtained in the encoding process and n_{BIN}^{a} is the number of additional binary segment mentioned in maintext. As shown in Supplementary Figure 2, the setting of random pair iteration affects n_{BIN}^{a}. Let the iteration time be 100 as described in maintext,

$$\frac{n_{\mathrm{BIN}}^{a}}{n_{\mathrm{BIN}}} \in (0, 0.032). \tag{12}$$

Based on equation (11) and the simulation values in (12), the interval of d_{ACTUAL} can be calculated as:

$$d_{\mathrm{ACTUAL}} \in \left(\frac{n_{\mathrm{BIN}} \times (l_{\mathrm{DNA}} - l_{\mathrm{INDEX}})}{\frac{(1 + 0.032) \times n_{\mathrm{BIN}}}{2} \times l_{\mathrm{DNA}}}, \frac{n_{\mathrm{BIN}} \times (l_{\mathrm{DNA}} - l_{\mathrm{INDEX}})}{\frac{n_{\mathrm{BIN}}}{2} \times l_{\mathrm{DNA}}}\right). \tag{13}$$

After simplification,

$$d_{\mathrm{ACTUAL}} \in \left(\frac{2 \times (l_{\mathrm{DNA}} - l_{\mathrm{INDEX}})}{1.032 \times l_{\mathrm{DNA}}}, 2 - \frac{l_{\mathrm{INDEX}}}{l_{\mathrm{DNA}}}\right). \tag{14}$$

Take the parameter used in the experiment for evaluation: $l_{\mathrm{PAYLOAD}} = 128$, $l_{\mathrm{INDEX}} = 16$, $\mathrm{HOMO} = 4$, $\mathrm{GCBIAS} = 0.1$. Thus, $l_{\mathrm{DNA}} = 128 + 16 = 144$. Assume $n_{\mathrm{BIN}} = 65536$, according to equation (4), f_{HOMO} is calculated to be 0.0486. According to equation (6), f_{GCBIAS} is calculated to be 0.0002. And according to equation (8), f_{INDEX} is calculated to be 0.2222.

Based on the above, the theoretical upper bound (d_{THEORY}), information density of a baseline coding scheme (d_{BASELINE}), and the actual information density of YYC (d_{ACTUAL}) can be calculated. Based on equation (9),

$$d_{\mathrm{THEORY}} = 2 - \max\{0.0486, 0.0002, 0.2222\} = 1.7778,$$

while using the equation (10),

$$d_{\text{BASELINE}} = 2 - \text{sum}\{0.0486, 0.0002, 0.2222\} = 1.7290.$$

Moreover, through the equation (14),

$$d_{\text{ACTUAL}} \in (1.7227, 1.7778).$$

Sometimes, information density is calculated without considering the effect of index. In this condition, based on the above parameters and simulation values, theoretical upper bound (d^i_{THEORY}), information density of a baseline coding scheme (d^i_{BASELINE}), and the actual performance of YYC (d^i_{ACTUAL}) can be calculated. Based on equation (9),

$$d^i_{\text{THEORY}} = 2 - \max\{0.0486, 0.0002\} = 1.9514$$

while using the equation (10),

$$d^i_{\text{BASELINE}} = 2 - \text{sum}\{0.0486, 0.0002\} = 1.9512.$$

Moreover, through the equation (14),

$$d^i_{\text{ACTUAL}} \in (1.9380, 2).$$

Supplementary Section 3: Command line steps to encode and decode the data

For reproducibility, we provide the step-by-step pseudocodes in an Ubuntu environment using Python 3.7.3:

Encoding

```
python

>>> from yyc import pipeline

>>> from yyc import scheme

>>> pipeline.encode(method=scheme.YYC(support_bases="A", base_reference=[0, 1, 0, 1], current_code_matrix=[[1, 1, 0, 0], [1, 0, 0, 1], [1, 1, 0, 0], [1, 1, 0, 0]], search_count=100, max_homopolymer=4, max_content=0.6), input_path="./files/Mona Lisa.jpg", output_path="./output/mona_lisa.dna", model_path="./output/yyc.pkl", need_index=True, need_log=True)
```

Decoding

```
python

>>> from yyc import pipeline

>>> pipeline.decode(model_path="./output/yyc.pkl",input_path="./output/mona_lisa.dna", output_path="./output/output_mona_lisa.jpg", has_index=True, need_log=True)
```

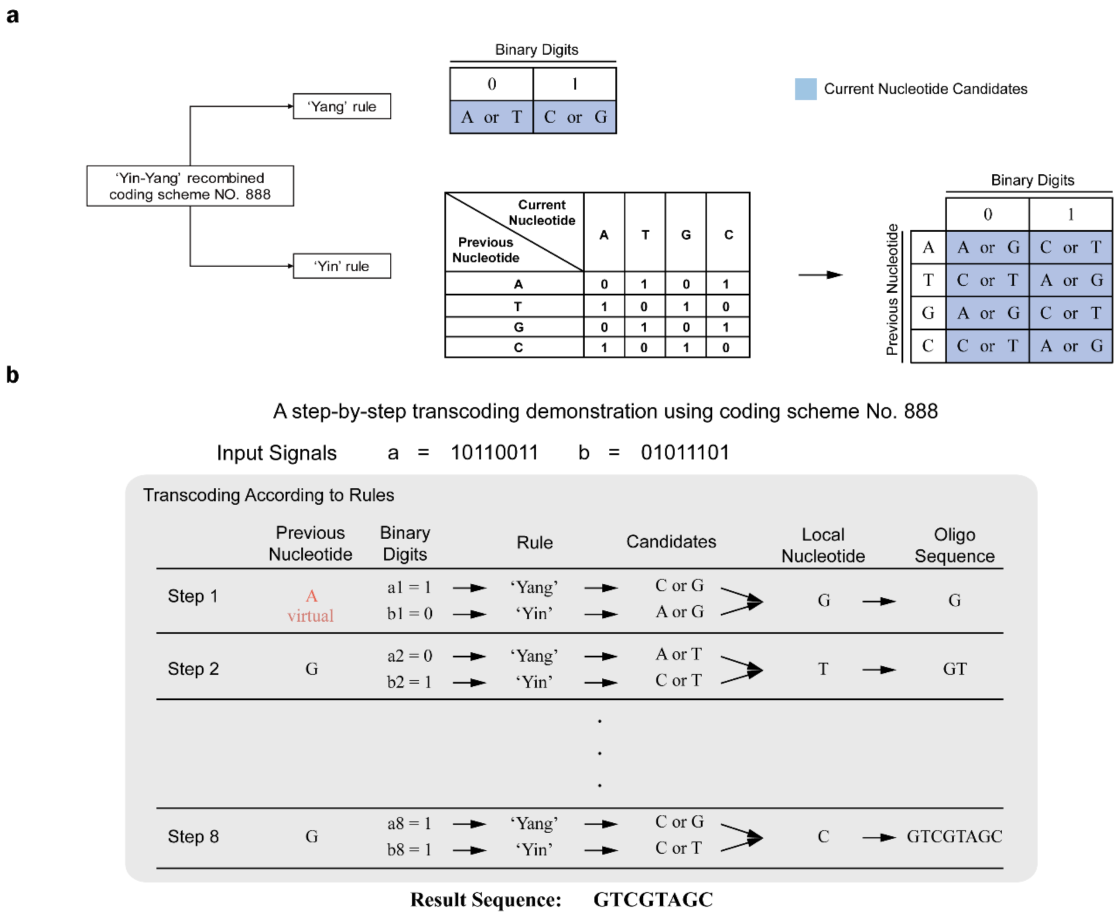

Supplementary Figure 1. The transcoding illustration using one of the 1536 coding schemes of YYC. (a) The coding principle of YYC coding scheme No. 888. (b) The demonstration of step-by step YYC transcoding process. 'a1', 'b1' represents the first-position binary digit in segment 'a' and 'b', and so on. Virtual base A means this base is used only for determination of the output base in the first run of transcoding and will not appear in the result sequence.

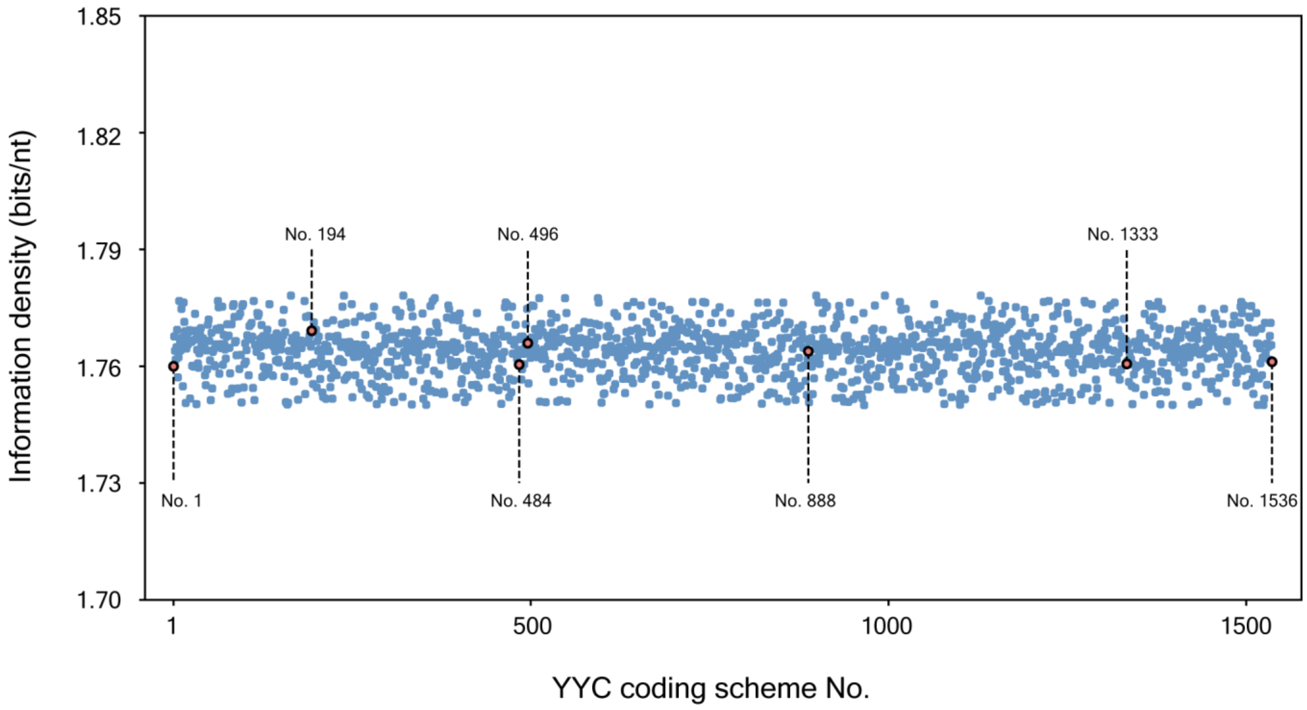

Supplementary Figure 2: Information density evaluation of 1536 YYC coding schemes using 1G data collection. Constraint parameters applied: maximum homopolymer run as 4, GC content 40%-60%, index length as 16, free energy of secondary structure > -30 kj/mol. YYC coding schemes NO. 1, 194, 484, 496, 888, 1333 and 1536 used in the simulation analysis and experimental validation are highlighted as red dots.

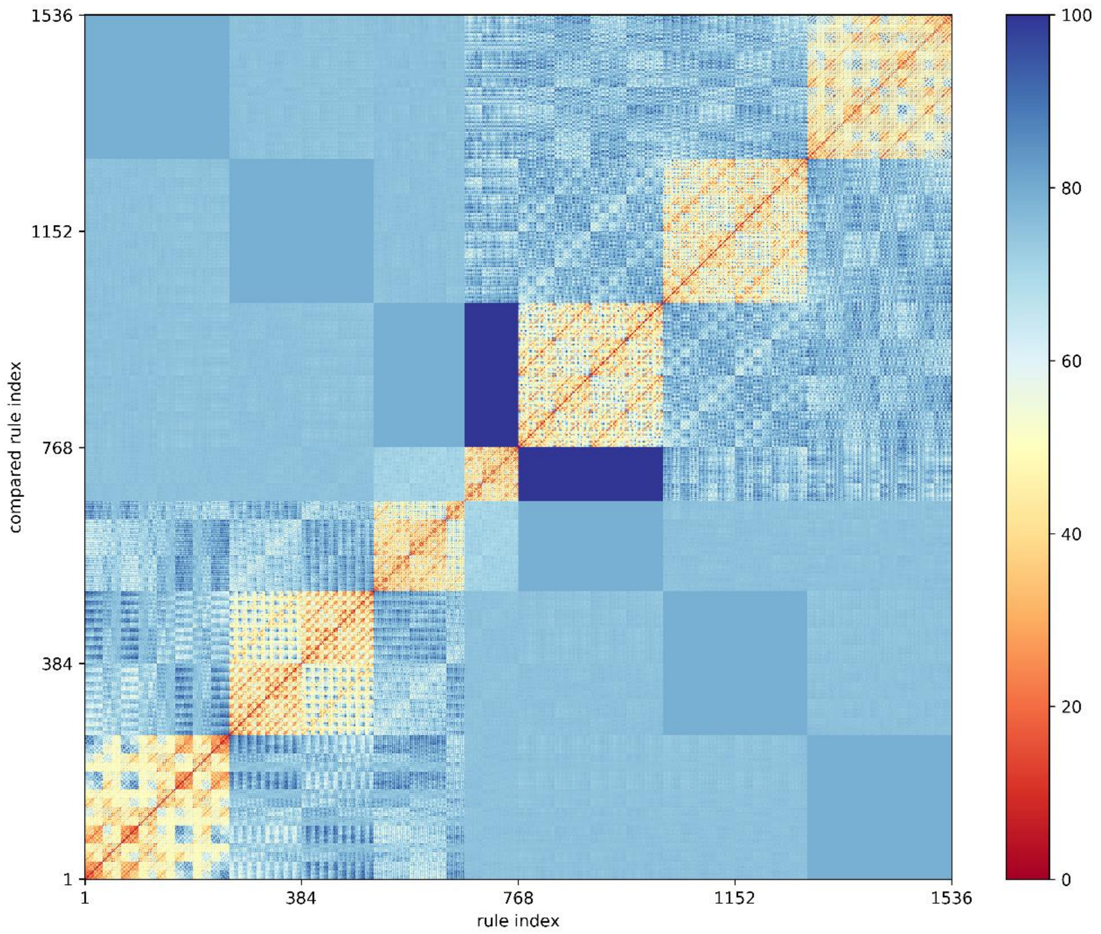

Supplementary Figure 3. Heatmap of average Hamming distance between sequences generated by 1,536 coding schemes. Colorbar shows difference between sequences generated by two coding schemes, calculated by (average hamming distance/sequence length × 100).

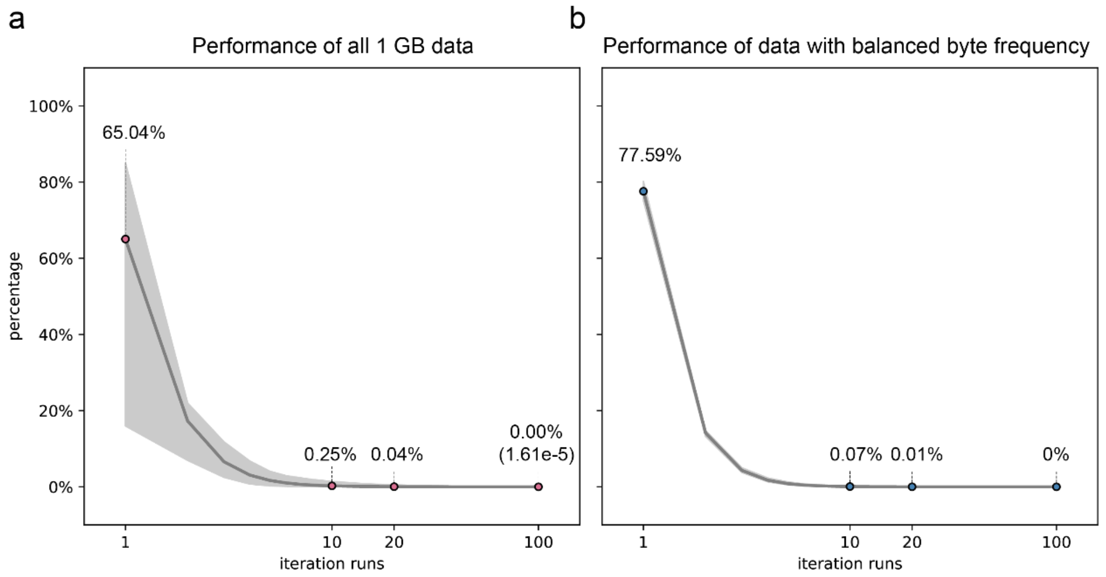

Supplementary Figure 4. Statistics of average iteration runs of 1 GB data encoding using all 1,536 rules. a) The performance of all the data, shadow represents percentage range for individual files. b) The performance of data with balanced byte frequency, the absolute count of iteration runs over 100 was 0.

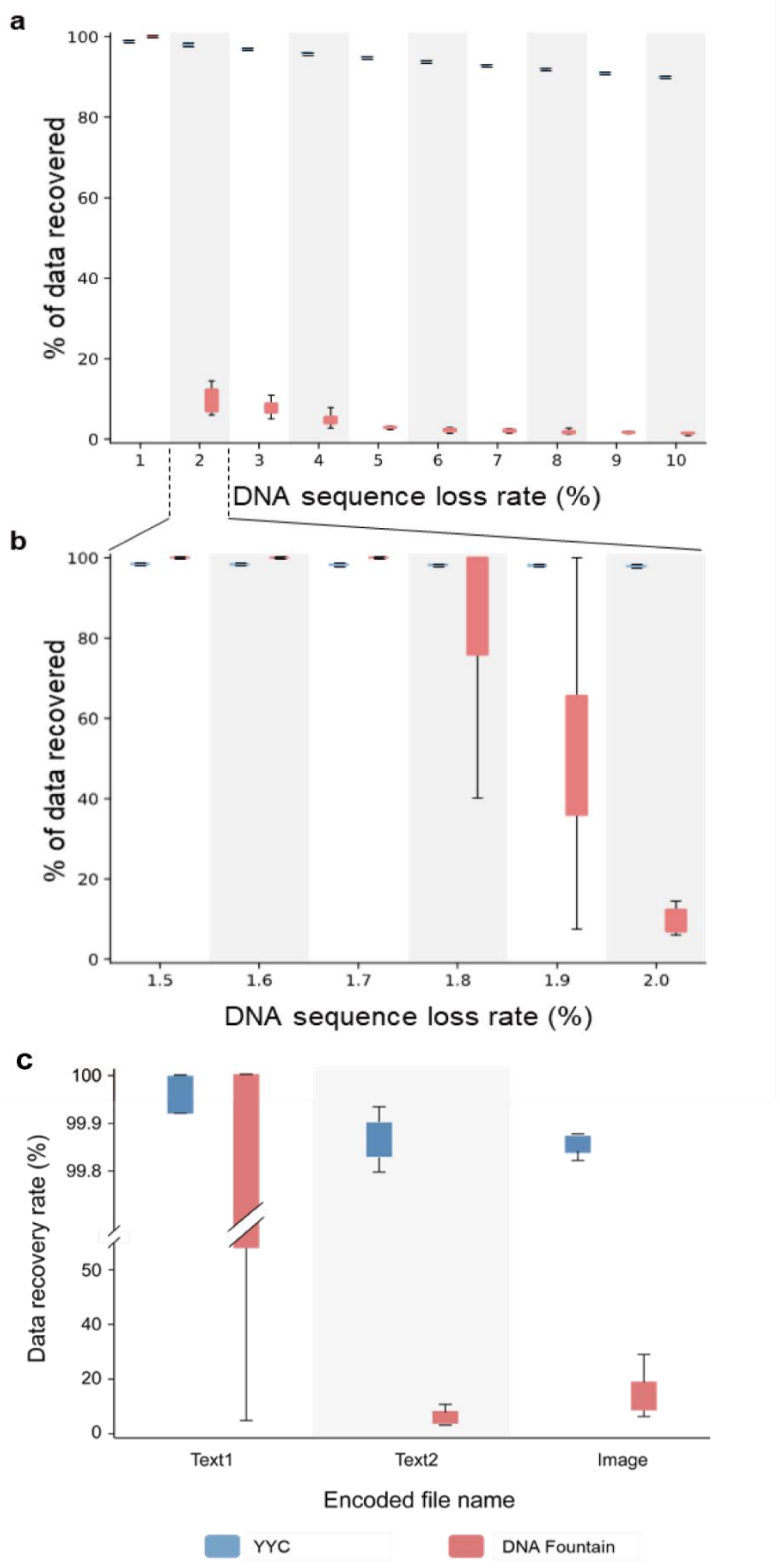

Supplementary Figure 5. The simulation analysis and experimental validation of YYC's data recovery rate in the presence of varying gradient of DNA sequence loss. *In-silico* simulation: *The lost rate between* **a)** *1% to 10% and* **b)** *1.5% to 2.0%.* **c)** *In vitro* experimental validation: *the data recovery rate of three files according to experimental validation result with average copy number of 1,000.* Data are presented in the form of box-and-whisker plot with maxima/minima and the bounds of boxes are first quartile to third quartile. (The sample sizes are 120 for panel a, 72 for panel b, 214 for panel c.)

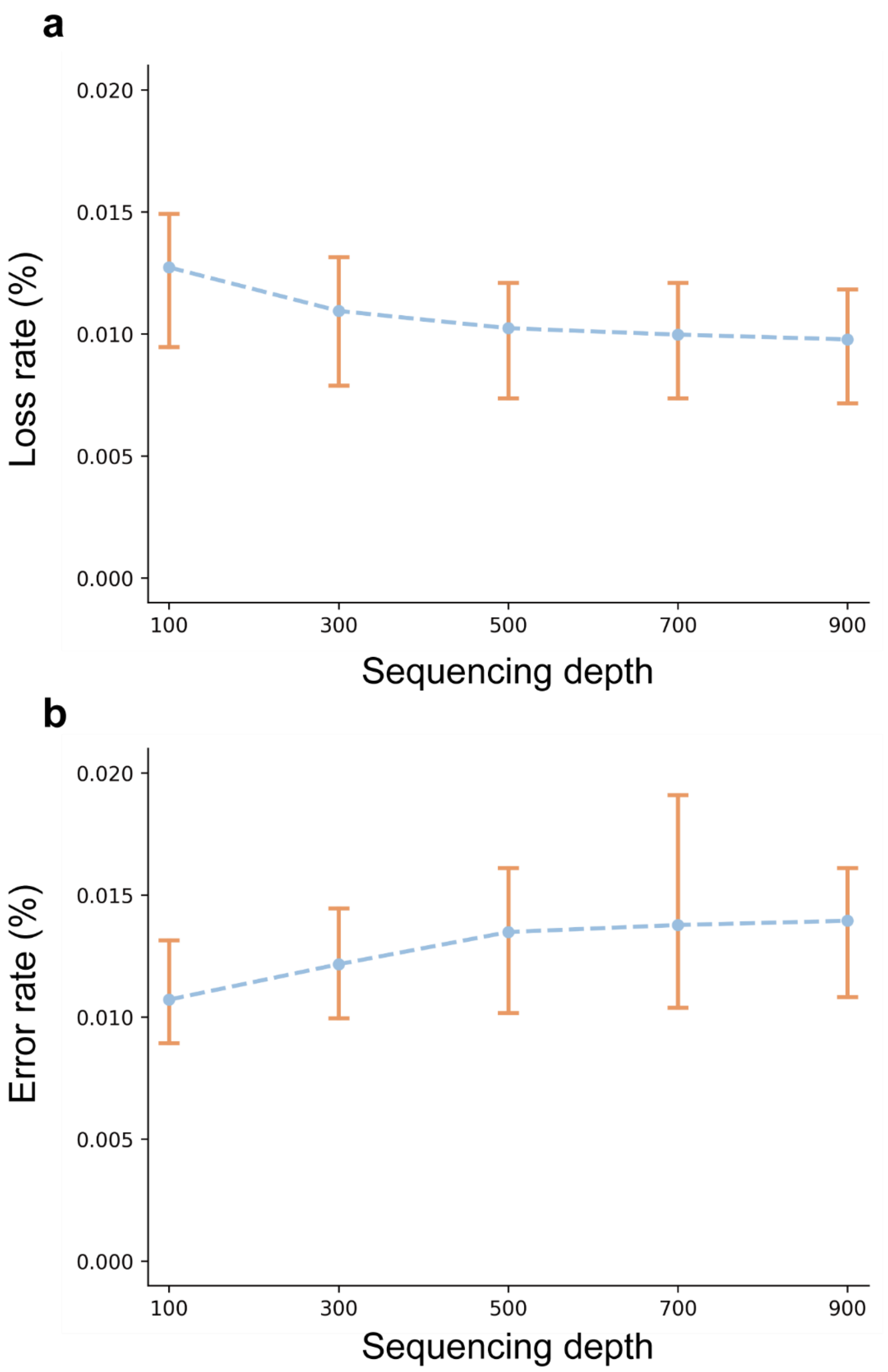

Supplementary Figure 6. Evaluation of the influence of sequencing depth on a) sequence error rate and b) sequence loss rate for *in vitro* storage validation. Blue: the average value of error rates of all samples under specific sequencing depth; Orange: The range of error rates of all samples under specific sequencing depth. Source data are available with the manuscript. (The sample size n=147 biologically independent experiments.)

Supplementary Table 1. The effect on compatibility of generated DNA sequence using different coding strategies for specific binary data patterns.

Binary Pattern (black = 1,white = 0)	GC content							Max homopolymer length						
	Church et. al	Goldman et. al	Grass et. al	DNA Fountain	Yin-Yang Code (No. 194)	Yin-Yang Code (No. 484)	Yin-Yang Code (No. 1333)	Church et. al	Goldman et. al	Grass et. al	DNA Fountain	Yin-Yang Code (No. 194)	Yin-Yang Code (No. 484)	Yin-Yang Code (No. 1333)
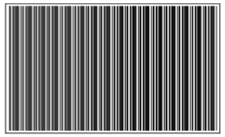				75.0%	44.53%	56.25%	57.03%				2	1	1	1
	48.83%	0.0%	44.44%	75.0%				3	1	1	2			
				0.0%	44.53%	56.25%	57.03%				64	1	1	1
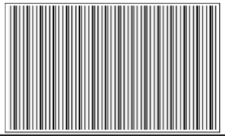				62.5%	44.53%	56.25%	56.25%				2	1	1	1
	51.95%	50.0%	0.0%	62.5%				3	1	2	2			
				0.0%	44.53%	56.25%	56.25%				64	1	1	1
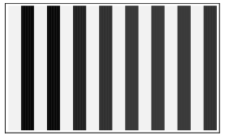				0.0%	50.00%	50.00%	50.00%				8	1	1	1
	51.56%	50.0%	55.56%	0.0%				3	1	3	8			
				0.0%	50.00%	50.00%	50.00%				64	1	1	1
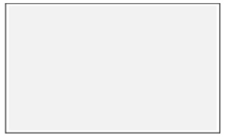				0.0%	50.00%	50.00%	50.00%				64	1	1	1
	50.0%	60.0%	33.33%	0.0%				3	1	2	64			
				0.0%	50.00%	50.00%	50.00%				64	1	1	1
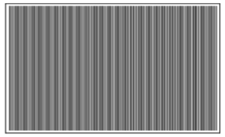				100.0%	50.00%	50.00%	50.78%				64	1	1	1
	51.56%	50.0%	22.22%	100.0%				1	1	2	64			
				0.0%	50.00%	50.00%	50.78%				64	1	1	1

Supplementary Table 2. Estimation number of coding scheme that can generate valid sequences for incorporating short binary sequences of one byte in varying range of 0/1 ratio. Constraint parameters: maximum homopolymer run as 4, GC content 40%-60%.

0/1 ratio	Number of coding schemes
0% - 5%	256
5%-10%	512
10%-15%	684
15%-20%	764
>20%	1536

Supplementary Table 3. The estimation of iteration runs and corresponding information density by transcoding 10 different types/formats of files.

File name	File format	File size (KB)	Rate of additional information (%)	Average number of iteration run	Information density (bits/nt) (Coding scheme No. 496)
Mona Lisa	jpg	96	0.031	1.398	1.8037
United Nations Flag	bmp	469	0.075	5.040	1.7764
A Tale of Two Cities	pdf	1011	0.072	1.825	1.7506
The Wandering Earth	pdf	368	0.064	1.924	1.7766
DNA Fountain Input Files	tar	2096	0.004	1.493	1.7391
Microsoft Winmine	exe	117	3.180	7.873	1.7489
For Elise	wma	2544	0.462	3.482	1.7311
Summer	mp3	6033	0.006	1.924	1.7265
Exiting the Factory	flv	4033	19.245	7.286	1.4480
I have a Dream	mp4	3986	0.094	1.923	1.7250

Supplementary Table 4. The estimation of information density and logical redundancy by DNA Fountain and YYC to varying file types. Net information density indicates the input information in bits divided by the nucleotides generated by coding schemes (excluding flanking primers). Minimum logical redundancy required for successful encoding and decoding larger than 100% is marked as 'N/A'. Bolded value indicates it is the best one among the tests.

**Type**	**Name**	**Net Information Density (bit/nt)**					**Logical Redundancy Percentage (%)**				
		DNA Fountain	YYC No.1	YYC No.496	YYC No.888	YYC No.1536	DNA Fountain	YYC No.1	YYC No.496	YYC No.888	YYC No.1536
Image	Mona Lisa.jpg	1.600	1.804	**1.805**	1.804	1.803	11.122	0.031	**0.000**	0.031	0.062
	United Nations Flag.bmp	0.920	1.775	**1.777**	1.776	1.774	93.134	0.175	**0.062**	0.125	0.237
Documents (Image + Text)	The Wandering Earth.pdf	1.654	1.776	1.777	**1.778**	1.777	7.501	0.120	0.032	**0.008**	0.032
	A Tale of Two Cities.pdf	1.684	1.750	1.750	1.751	**1.755**	5.560	0.078	0.079	0.073	**0.005**
Binary	DNA Fountain Input Files.tar.gz	1.723	1.739	1.739	1.739	**1.740**	3.204	0.008	0.007	0.006	**0.003**
	Microsoft Winmine.exe	1.441	1.719	**1.758**	1.729	1.752	23.371	4.982	**2.629**	3.854	2.979
Audio	For Elise.wma	1.715	1.730	**1.731**	1.731	1.731	3.686	0.549	**0.485**	0.487	0.447
	Summer.mp3	1.725	1.726	1.727	**1.727**	1.726	3.069	0.025	0.004	**0.003**	0.025
Video	Exiting the Factory.flv	1.679	1.440	1.449	**1.719**	1.443	5.908	19.875	17.266	**2.514**	19.211
	I have a Dream.mp4	1.722	**1.726**	1.725	1.725	1.725	3.268	**0.087**	0.090	0.097	0.091

Supplementary Table 5. The *in-silico* simulation of the data recovery rate in the context of a gradient of DNA sequence loss.

**Oligo loss rate**	**YYC**	**DNA Fountain**
1.0%	99.00% ± 0.00%	100.00% ± 0.00%
1.5%	98.50% ± 0.00%	100.00% ± 0.00%
1.6%	98.40% ± 0.00%	100.00% ± 0.00%
1.7%	98.30% ± 0.00%	100.00% ± 0.00%
1.8%	98.20% ± 0.00%	84.66% ± 23.11%
1.9%	98.10% ± 0.00%	53.20% ± 29.06%
2.0%	98.00% ± 0.00%	9.82% ± 3.17%
3.0%	97.00% ± 0.00%	7.84% ± 1.90%
4.0%	96.00% ± 0.00%	4.82% ± 1.66%
5.0%	95.00% ± 0.00%	2.9% ± 0.27%
6.0%	94.00% ± 0.00%	2.18% ± 0.50%
7.0%	93.00% ± 0.00%	2.04% ± 0.39%
8.0%	92.00% ± 0.00%	1.76% ± 0.55%
9.0%	91.00% ± 0.00%	1.68% ± 0.18%
10.0%	90.00% ± 0.00%	1.42% ± 0.28%

Supplementary Table 6. Minimum redundancy required for successful data retrieval with different bitmap images. Minimum required redundancy for successful encoding and decoding larger than 300% is marked as 'N.A.'.

Name	Flag Style	Minimum Available Redundancy
China Flag	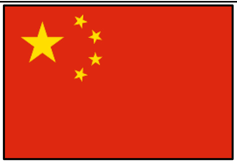	> 243%
Japan Flag	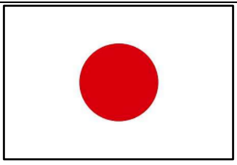	N.A.
India Flag	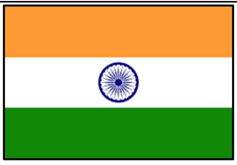	> 42%
Britain Flag	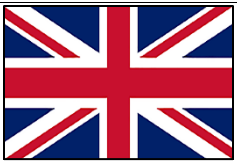	13%
Ireland Flag	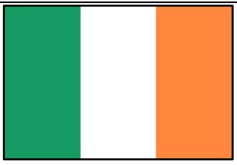	N.A.
Germany Flag	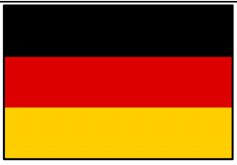	N.A.
America Flag	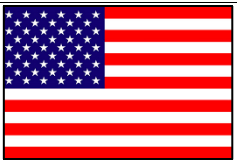	> 61%
Brazil Flag	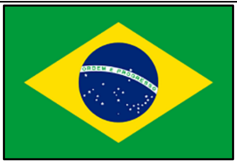	> 116%
Singapore Flag	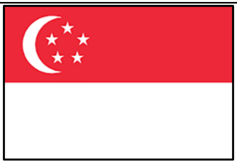	33%

Supplementary Table 7. Error analysis of data coding DNA in plasmid pRS416 for *in vivo* storage demonstration. Sequences are mapping to original sequence. SNV refers to substitution of a single nucleotide. Indel refers to insertion and deletion of a single nucleotide. In this study, only deletion was observed for structural variation.

Strain ID	Single nucleotide variation (SNV)	1-nt Indel	Structural variation (SV)
L01	140	26	4
L02	146	41	2
L03	122	37	1
L04	23	6	14
L05	138	28	3
L06	141	33	2
L07	127	35	3
L08	143	37	2
L09	151	26	2
L10	132	40	2
L11	113	22	2
L12	136	34	3
L13	133	39	2
L14	124	32	2
L15	155	26	1
L16	122	29	1

References:

[1] Shannon, C. E. (1948). A mathematical theory of communication. The Bell system technical journal, 27(3), 379-423.

[2] Nguyen, T. T., Cai, K., Immink, K. A. S., & Kiah, H. M. (2021). Capacity-Approaching Constrained Codes with Error Correction for DNA-Based Data Storage. IEEE Transactions on Information Theory.

[3] Jech, T. (2013). Set theory. Springer Science & Business Media.
