# Supplementary material for: Towards practical and robust DNA-based data archiving using the yin–yang codec system
Source: Nat Comput Sci. 2022 Apr 25;2(4):234–42. doi: 10.1038/s43588-022-00231-2 (PMC10766522; doi:10.1038/s43588-022-00231-2)
Supplement: Supplementary file 2 — Reporting Summary [file 43588_2022_231_MOESM2_ESM.pdf]

nature research

Corresponding author(s): Xun Xu
George M. Church
Yue Shen

Last updated by author(s): Mar 16, 2022

Reporting Summary

Nature Research wishes to improve the reproducibility of the work that we publish. This form provides structure for consistency and transparency in reporting. For further information on Nature Research policies, see our Editorial Policies and the Editorial Policy Checklist.

Statistics

For all statistical analyses, confirm that the following items are present in the figure legend, table legend, main text, or Methods section.

n/a	Confirmed	
☐	☒	The exact sample size (*n*) for each experimental group/condition, given as a discrete number and unit of measurement
☐	☒	A statement on whether measurements were taken from distinct samples or whether the same sample was measured repeatedly
☐	☒	The statistical test(s) used AND whether they are one- or two-sided<br>*Only common tests should be described solely by name; describe more complex techniques in the Methods section.*
☐	☒	A description of all covariates tested
☐	☒	A description of any assumptions or corrections, such as tests of normality and adjustment for multiple comparisons
☐	☒	A full description of the statistical parameters including central tendency (e.g. means) or other basic estimates (e.g. regression coefficient) AND variation (e.g. standard deviation) or associated estimates of uncertainty (e.g. confidence intervals)
☒	☐	For null hypothesis testing, the test statistic (e.g. F, t, r) with confidence intervals, effect sizes, degrees of freedom and P value noted<br>*Give P values as exact values whenever suitable.*
☒	☐	For Bayesian analysis, information on the choice of priors and Markov chain Monte Carlo settings
☒	☐	For hierarchical and complex designs, identification of the appropriate level for tests and full reporting of outcomes
☐	☒	Estimates of effect sizes (e.g. Cohen's *d*, Pearson's *r*), indicating how they were calculated

Our web collection on statistics for biologists contains articles on many of the points above.

Software and code

Policy information about availability of computer code

Data collection	We published our custom python codes on Github and Zenodo. Source code and user manual are also available under: https://github.com/ntpz870817/DNA-storage-YYC and https://doi.org/10.5281/zenodo.6326563.
Data analysis	All encoding, decoding, and error analyzing experiments were performed in an Ubuntu 16.04.7 environment including an i7 CPU and 16 GB of RAM using Python 3.7.3, with our developed package "Chamaeleo" available under: https://github.com/ntpz870817/Chamaeleo.<br>For NGS result analysis, we used BWA v0.7.13, samtools v0.1.19-44428cd and vSOAP 2.7.7. For multiple sequence alignment, we used clustalW2 (CLUSTAL v2.1).

For manuscripts utilizing custom algorithms or software that are central to the research but not yet described in published literature, software must be made available to editors and reviewers. We strongly encourage code deposition in a community repository (e.g. GitHub). See the Nature Research guidelines for submitting code & software for further information.

Data

Policy information about availability of data

All manuscripts must include a data availability statement. This statement should provide the following information, where applicable:

- Accession codes, unique identifiers, or web links for publicly available datasets
- A list of figures that have associated raw data
- A description of any restrictions on data availability

The data that support the findings of this study have been deposited in the CNSA (https://db.cngb.org/cnsa/) of CNGBdb with accession code CNP0001650.
The figures-associated raw data is provided with the paper.
No restriction is on data availability.

Field-specific reporting

Please select the one below that is the best fit for your research. If you are not sure, read the appropriate sections before making your selection.

☒ Life sciences ☐ Behavioural & social sciences ☐ Ecological, evolutionary & environmental sciences

For a reference copy of the document with all sections, see nature.com/documents/nr-reporting-summary-flat.pdf

Life sciences study design

All studies must disclose on these points even when the disclosure is negative.

Sample size	'Sample size' was determined by technical properties of DNA synthesis and sequencing. Different kinds and formats of data were chosen for DNA-based data storage, which is sufficient to prove the universality and robustness of this codec.
Data exclusions	Raw sequencing data was filtered under a pre-established criteria, 10% low-quality reads (Phred score < 20) by SOAPnuke were filtered.
Replication	The source data retrieval experiments were repeated twice for two batches of synthesized oligo pools. The attempts were always successful for the replications. The original data was successfully decoded in all technical repeats, or replications. The experiments of second batch were conducted about one year after the those of first batch.
Randomization	The simulations were run with precisely defined parameter value settings. Experimental validation also used precisely defined sequences and standard operation. Thus, randomization is not relevant to the study.
Blinding	The simulations were run with precisely defined parameter value settings. Experimental validation also used precisely defined sequences and standard operation. Thus, blinding is not relevant to the study.

Reporting for specific materials, systems and methods

We require information from authors about some types of materials, experimental systems and methods used in many studies. Here, indicate whether each material, system or method listed is relevant to your study. If you are not sure if a list item applies to your research, read the appropriate section before selecting a response.

Materials & experimental systems

n/a	Involved in the study	
☒	☐	Antibodies
☒	☐	Eukaryotic cell lines
☒	☐	Palaeontology and archaeology
☒	☐	Animals and other organisms
☒	☐	Human research participants
☒	☐	Clinical data
☒	☐	Dual use research of concern

Methods

n/a	Involved in the study	
☒	☐	ChIP-seq
☒	☐	Flow cytometry
☒	☐	MRI-based neuroimaging
